# fMRI-Targeted High-Angular Resolution Diffusion MR Tractography to Identify Functional Language Tracts in Healthy Controls and Glioma Patients

**DOI:** 10.3389/fnins.2020.00225

**Published:** 2020-03-31

**Authors:** Francesco Sanvito, Eduardo Caverzasi, Marco Riva, Kesshi M. Jordan, Valeria Blasi, Paola Scifo, Antonella Iadanza, Sofia Allegra Crespi, Sara Cirillo, Alessandra Casarotti, Antonella Leonetti, Guglielmo Puglisi, Marco Grimaldi, Lorenzo Bello, Maria Luisa Gorno-Tempini, Roland G. Henry, Andrea Falini, Antonella Castellano

**Affiliations:** ^1^Neuroradiology Unit and CERMAC, IRCCS San Raffaele Scientific Institute, Milan, Italy; ^2^School of Medicine, Vita-Salute San Raffaele University, Milan, Italy; ^3^UCSF Weill Institute for Neurosciences, Department of Neurology, University of California, San Francisco, San Francisco, CA, United States; ^4^Department of Medical Biotechnology and Translational Medicine, Università degli Studi di Milano, Milan, Italy; ^5^Neurosurgical Oncology Unit, Humanitas Clinical and Research Center – IRCCS, Rozzano, Italy; ^6^IRCCS Fondazione Don Carlo Gnocchi, Milan, Italy; ^7^Nuclear Medicine Unit, IRCCS San Raffaele Scientific Institute, Milan, Italy; ^8^School of Psychology, Vita-Salute San Raffaele University, Milan, Italy; ^9^Neuroradiology Unit, Humanitas Clinical and Research Center – IRCCS, Rozzano, Italy; ^10^Department of Oncology and Hemato-Oncology, Università degli Studi di Milano, Milan, Italy

**Keywords:** tractography, high angular resolution diffusion imaging, brain tumor, fMRI, task-fMRI, language network, presurgical brain mapping

## Abstract

**Background:**

MR Tractography enables non-invasive preoperative depiction of language subcortical tracts, which is crucial for the presurgical work-up of brain tumors; however, it cannot evaluate the exact function of the fibers.

**Purpose:**

A systematic pipeline was developed to combine tractography reconstruction of language fiber bundles, based on anatomical landmarks (Anatomical-T), with language fMRI cortical activations. A fMRI-targeted Tractography (fMRI-T) was thus obtained, depicting the subsets of the anatomical tracts whose endpoints are located inside a fMRI activation. We hypothesized that fMRI-T could provide additional functional information regarding the subcortical structures, better reflecting the eloquent white matter structures identified intraoperatively.

**Methods:**

Both Anatomical-T and fMRI-T of language fiber tracts were performed on 16 controls and preoperatively on 16 patients with left-hemisphere brain tumors, using a *q*-ball residual bootstrap algorithm based on High Angular Resolution Diffusion Imaging (HARDI) datasets (*b* = 3000 s/mm^2^; 60 directions); fMRI ROIs were obtained using picture naming, verbal fluency, and auditory verb generation tasks. In healthy controls, normalized MNI atlases of fMRI-T and Anatomical-T were obtained. In patients, the surgical resection of the tumor was pursued by identifying eloquent structures with intraoperative direct electrical stimulation mapping and extending surgery to the functional boundaries. Post-surgical MRI allowed to identify Anatomical-T and fMRI-T non-eloquent portions removed during the procedure.

**Results:**

MNI Atlases showed that fMRI-T is a subset of Anatomical-T, and that different task-specific fMRI-T involve both shared subsets and task-specific subsets – e.g., verbal fluency fMRI-T strongly involves dorsal frontal tracts, consistently with the phonogical-articulatory features of this task. A quantitative analysis in patients revealed that Anatomical-T removed portions of AF-SLF and IFOF were significantly greater than verbal fluency fMRI-T ones, suggesting that fMRI-T is a more specific approach. In addition, qualitative analyses showed that fMRI-T AF-SLF and IFOF predict the exact functional limits of resection with increased specificity when compared to Anatomical-T counterparts, especially the superior frontal portion of IFOF, in a subcohort of patients.

**Conclusion:**

These results suggest that performing fMRI-T in addition to the ‘classic’ Anatomical-T may be useful in a preoperative setting to identify the ‘high-risk subsets’ that should be spared during the surgical procedure.

## Introduction

The accurate identification of eloquent fiber tracts, such as language bundles, is a crucial step in the surgical work-up of brain tumors. In fact, the treatment goal for these neoplasms is the maximal safe resection (Sanai et al., 2008; Yordanova et al., 2011; Duffau, 2016), preserving eloquent structures underlying fundamental neurological functions such as language, vision, and motor skills (Duffau, 2012).

The gold standard technique to achieve the correct localization of these structures is the cortical and subcortical direct electrical stimulation (DES) performed during the surgical procedure (Ojemann, 1983; Berger et al., 1990; Berger and Ojemann, 1992). Nevertheless, DES is an invasive technique that requires the patient to perform language tasks while awake (awake-surgery) after craniotomy.

Currently, MR Tractography is the only method that can depict fiber tract localization and their relationship with the lesion *before* the craniotomy and non-invasively. Based on diffusion-weighted imaging that reflects water diffusion features in biological tissues, this technique allows to infer fiber trajectory, since water diffusion in the white matter is preferentially oriented along the direction of the axonal fibers (Mori and Zhang, 2006; Mukherjee et al., 2008). MR Tractography enables the *in vivo* non-invasive depiction of subcortical fascicles and it has thus rapidly become fundamental in the presurgical assessment of brain tumors (Bello et al., 2010; Riva et al., 2011; Castellano et al., 2012; Ulmer et al., 2014), in order to evaluate the displacement or modifications of the eloquent bundles before performing brain surgery (Bello et al., 2008; Bizzi, 2009; Essayed et al., 2017), to predict the extent of resection (Castellano et al., 2012), and to better tailor the extent of the craniotomy (Romano et al., 2009). In addition, MR Tractography reconstructions can be loaded onto the neuronavigational system in order to guide DES during awake surgery, thus decreasing the duration of surgery, patient fatigue, and intraoperative seizures (Bertani et al., 2009; Riva et al., 2011; Castellano et al., 2017).

Although the original Tractography approaches based on Diffusion Tensor Imaging (DTI) (Mori et al., 1999; Jellison et al., 2004; Stadlbauer et al., 2007) are still used in the clinics, new algorithms based on High Angular Resolution Diffusion Imaging (HARDI) acquisitions (Tournier et al., 2004; Tuch, 2004; Hess et al., 2006) – such as the residual-bootstrap q-ball algorithm – have brought several improvements to this technique in terms of sensibility and accuracy (Bucci et al., 2013) and have already been proven as clinically feasible (Caverzasi et al., 2015).

Nevertheless, Tractography provides information exclusively about the anatomical trajectory of white matter fascicles, regardless of their function. In fact, recent evidence has demonstrated that some components of language bundles identified with MR Tractography can be safely removed intraoperatively, since they do not elicit transient deficit when stimulated by means of DES (Mandonnet et al., 2007; Bello et al., 2008).

In the presurgical setting, data about brain functions can be provided by functional MRI (fMRI), that employs BOLD (blood oxygenation level dependent) contrast to map cortical activity (Bizzi, 2009; Castellano et al., 2017). However, this technique enables to identify exclusively cortical areas related with a given task, and cannot detect subcortical structures.

In this study, we hypothesized that combining Tractography (an anatomy-based technique) with fMRI (a function-based technique) will provide additional information about the subsets of the anatomical subcortical tracts that are more likely to be eloquent for the language function.

We develop a systematic pipeline to combine the “classic” Tractography of language bundles, based exclusively on anatomical landmarks (Anatomical-T), with language fMRI cortical activations, in order to achieve a fMRI-targeted Tractography (fMRI-T) that depicts the subsets of the anatomical tracts whose endpoints are located inside a fMRI activation.

In order to test this hypothesis, we performed Anatomical-T and fMRI-T and evaluated their relationship with the DES-based limits of surgical resection. By providing this additional functional information, fMRI-T may contribute to depict the physiological functional subcortical network underlying each task in the healthy controls’ cohort, and the “high-risk subsets” of the subcortical bundles that should be spared during the surgical procedure in the patients’ cohort.

## Materials and Methods

### Subjects

Healthy controls’ cohort included 16 right-handed native Italian speakers (7 men, 9 women; mean age, 29 years; range, 21-48 years). None of these individuals had a history of neurological disorders and their brain MRI scans presented no abnormalities.

Patients’ cohort included 16 patients with left-hemisphere presumed gliomas (14 men, 2 women; mean age, 36 years; range, 18-68 years) retrospectively selected among those scanned at our Institution. Inclusion criteria were as follows: tumor resection based on awake-surgery guided by cortical and subcortical DES, availability of pre-surgical HARDI and language task-based fMRI datasets, availability of post-surgical conventional MRI, no history of other neurological disorders. Tumor histotypes included mainly grade II and grade III astrocytomas and oligodendrogliomas, none of these patients presented with glioblastoma. Surgical gross total resection (GTR, 100% extent of resection) had been achieved in 10 patients, whereas in 6 patients only a subtotal resection (STR) was possible, with extents of resection ranging from 81 to 98%.

All subjects gave informed consent to have their data used for research purpose.

### MRI Acquisition Protocol

MRI acquisitions were performed at 3.0 T (Philips Achieva – Philips Healthcare, Best, Netherlands).

Conventional MRI protocol included axial T2-weighted Turbo-spin-echo (TSE) images (TR/TE 3000/85 ms; flip angle, 90°; Field of View [FOV], 230 mm; 22 slices; thickness, 5/1 mm gap; matrix, 512 × 512; SENSitivity-Encoding [SENSE] reduction factor, *R* = 1.5; acquisition time, 3 min 42 s), axial 3D Fluid Attenuated Inversion Recovery (3D-FLAIR) images (TR/TE/TI 10000/110/2750 ms; flip angle, 90°; FOV, 230 mm; 90 slices; thickness, 1.5/0 mm gap; matrix, 224 × 256; SENSE reduction factor *R* = 2; acquisition time 8 min 20 s), and axial T1-weighted Fast Field Echo (FFE) Multi-Shot images (TR/TE 8/4 ms; flip angle, 8°; FOV, 240 mm; 56 slices; thickness, 2.5/0 mm gap; matrix 256 × 256; SENSE reduction factor, *R* = 1; acquisition time, 1 min 46 s).

High Angular Resolution Diffusion Imaging (HARDI) data were obtained using an axial Single-Shot Spin-Echo Echo Planar Imaging (EPI) sequence and diffusion gradients were applied along 60 non-collinear directions (*b*-value, 3000 s/mm^2^; TR/TE, 12000/74 ms; SENSE reduction factor, *R* = 2; in-plane resolution, 1.87 × 1.87 mm^2^; 50 slices; thickness, 2.5/0 mm gap; FOV, 240 mm; matrix, 128 × 128; acquisition time, 13 min).

Three covert language tasks were employed for language fMRI: Picture Naming (PN), Verbal Fluency (VF), Auditory Verb Generation (AVG).

Picture Naming (PN): naming a series of common objects (i.e., “chair,” “house,” and “knife”) presented on a screen; baseline consisted in looking at scrambled non-sense figures.

Verbal Fluency (VF): listing as many nouns starting with a given letter presented aurally (i.e., “fork,” “field,” “foot”; when the subject hears the letter “F”); baseline consisted in repeatedly counting from 1 to 10.

Auditory Verb Generation (AVG): generating an appropriate verb from a given noun presented aurally (i.e., “to eat”; when the subject hears the noun “bread”); baseline consisted in repeatedly counting from 1 to 10.

All healthy controls fulfilled all three tasks, patients’ fMRI employed generally two tasks (with the exceptions of three patients, two of whom performed just one task, and one of whom performed all three tasks). For each patient, fMRI tasks were selected as follows: PN fMRI was acquired for every patient due to a neurosurgeons’ preference (since intraoperative DES included a picture naming task), verbal VF fMRI was mainly reserved to patients with frontal tumors (in order to assess the relationship between fMRI activations and frontal phonological-articulatory areas, 9 patients), AVG fMRI was mainly applied to patients with temporal or parietal lesions (in order to map fMRI activations in the Wernicke area, 6 patients). In some cases, VF or AVG were not performed due to clinical scanning time constraints and/or suboptimal patient’s collaboration.

Block-design task-based fMRI data were based on BOLD (blood oxygenation level dependent) contrast obtained using T2^∗^-weighted Gradient-Echo EPI (GE-EPI) sequences (TR/TE, 3700/30 ms; TE, 30 ms; flip angle, 85°; FOV, 240 mm; matrix, 128 × 128; in-plane resolution 1.87 × 1.87 mm^2^; 32 slices; thickness, 4 mm; SENSE factor *R* = 2; 80 dynamic scans for PN task, 100 for VF task, 100 for AVG task).

3.0 T post-surgical 3D-FLAIR images were acquired 24/48 h after the procedure.

### fMRI Subject-Level Analysis

fMRI data obtained from both healthy controls and patients were analyzed with SPM8 (Wellcome Dept. Cogn. Neurol., London^1^) using MATLAB 7.1 (MathWork, Natick, MA, United States). Analyses were performed following a standard processing pipeline (Bizzi et al., 2008).

Images were corrected for motion, realigned to the mean image, and the estimated movement parameters were used as regressors in a single-subject statistical analysis. These realigned images were then spatially smoothed using a 8-mm full-width at half-maximum isotropic Gaussian kernel. The expected hemodynamic response function of the software package was modeled with a block design. In every subject, a *t*-contrast was defined according to the General Linear Model for each task: verbal fluency, auditory verb generation and picture naming. Activations were considered significant if survived *P* < 0.001 uncorrected statistical threshold.

### HARDI Preprocessing and Tractography Algorithm

Movement and eddy-current distortions were corrected using the FMRIB Software Library (University of Oxford^2^), then the original gradient table was consequently rotated using the FSL “fdt rotate bvecs” function. Diffusion tensor and fractional anisotropy (FA) maps were estimated using Diffusion imaging in Python (Dipy) software (Soares et al., 2013; Garyfallidis and Brett, 2014).

Tractography was based on a q-ball residual bootstrap algorithm (Berman et al., 2008; Caverzasi et al., 2014, 2015), following the steps described by Caverzasi et al. (2015) in order to fit the signal to spherical harmonics, to compute the Orientation Distribution Functions (ODFs), and to identify the primary and principal fiber orientations. The tracking was seeded from tract-specific seed-ROIs, as described in the following paragraph (2.5). Maximum turning angle of 60° (Caverzasi et al., 2015) and FA threshold of 0.10 (Bello et al., 2008) were used as stopping criteria. Seed density was set at 7^3^ per voxel for healthy controls, and at 9^3^ per voxel for patients, in order to compensate for streamline loss in the patients’ cohort due to FA drops within the tissue affected by edema or tumor infiltration. Such compensation in the patients’ cohort was necessary in order to obtain a sufficient number of streamlines that were able to survive the FA stopping criteria and reach the cortex, allowing the subsequent fMRI-targeting operation.

### Anatomical-T

Anatomical-T approach was used to depict 8 left-hemisphere language tracts for each subject, including dorsal and ventral tracts (Supplementary Figure 1).

Dorsal tracts: Frontal Aslant Tract (FAT); Arcuate Fasciculus (AF) or long segment of the perisylvian language network; Superior Longitudinal Fasciculus component II (SLF-II), SLF component III (SLF-III), or anterior segment of the perisylvian language network; temporoparietal component of SLF (SLF-tp) or posterior segment of the perisylvian language network.

Ventral tracts: Inferior Fronto-Occipital Fasciculus (IFOF), Uncinate Fasciculus (UF), Inferior Longitudinal Fasciculus (ILF).

Anatomical-T for all tracts but FAT employed regions of interest (ROIs) placed exclusively referring to the anatomical seed-ROIs and target-ROIs landmarks reported by Caverzasi et al. (2015). As for FAT, a seed-ROI was placed on an axial plane including the subcortical voxels of the superior frontal gyrus corresponding to the supplementary motor area, and the inferior frontal gyrus was used as a target-ROI – these ROIs were chosen on the basis of the current knowledge regarding FAT trajectory (Dick et al., 2014).

Results were visualized using Trackvis^3^ and carefully inspected in order to quality-check the tracts and to remove all the following: obvious artifacts, streamlines directed toward the basal ganglia, the pons, and the right hemisphere.

### fMRI-T Tract Generation Pipeline

fMRI-targeted tractography tracts were generated by selecting the streamlines of Anatomical-T tracts that reached a task-specific fMRI-activated cortical area. Their generation was based on a two-step pipeline: the first step consisted in obtaining a fMRI-target-ROI that reflected the subject-specific task-specific activation areas; the second step consisted in generating fMRI-T tracts representing the subsets of the Anatomical-T tracts whose endpoints are located inside the fMRI-target-ROI.

#### Step 1

A binary fMRI-ROI was obtained for each subject’s task by binarizing the subject-level fMRI results, and then registered to the corresponding subject’s diffusion space using the FSL linear registration FLIRT tool (University of Oxford^2^). The relationship between this fMRI-ROI and the Anatomical-T tracts was evaluated using Trackvis^3^ in order to decide whether or not some manual corrections (extension and/or reduction) were needed in order to obtain the definitive fMRI-target-ROI. These manual corrections were applied only in case certain criteria were met, which were strictly determined *a priori* by three of the authors (FS, AnC, EC) as follows.

A manual extension of the fMRI-ROI was performed exclusively when the endpoints of some streamlines were located within 3 voxels outside the fMRI-ROI and in the same anatomical area (same gyrus or sulcus) – a similar ROI-extension correction is also reported in other fMRI-based Tractography studies (Upadhyay et al., 2007; Zhu et al., 2014).

A manual reduction of the fMRI-ROI was performed exclusively when the endpoints of some streamlines were located in a non-cortical part of the fMRI-ROI (e.g., basal ganglia) or in a part of the fMRI-ROI belonging to a different lobe (e.g., temporal pole streamlines terminating in inferior frontal or insular part of the fMRI-ROI).

#### Step 2

**FIGURE 1:**
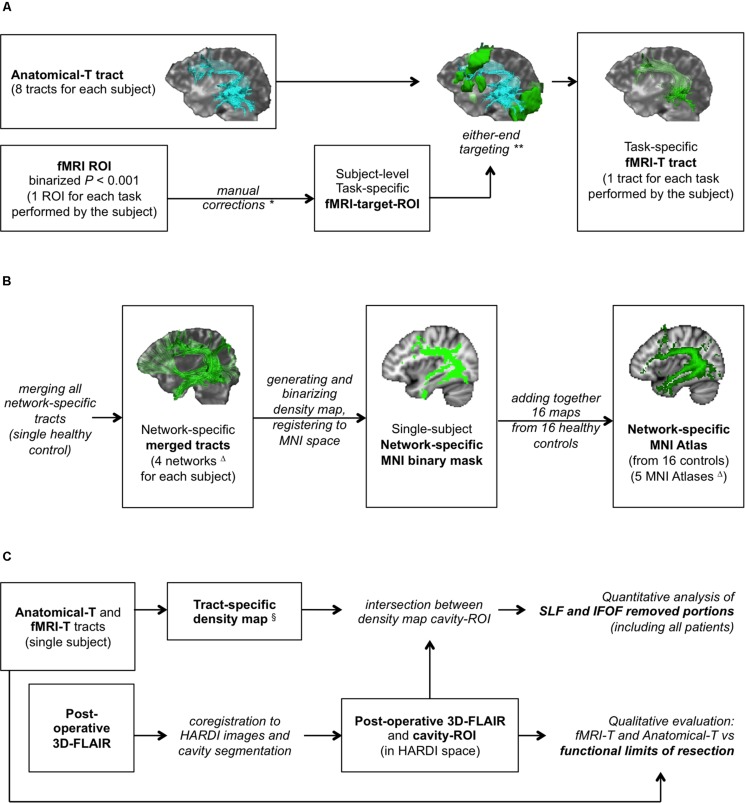
Study design. **(A)** fMRI-targeted Tractography (fMRI-T) tracts generation pipeline (applied to both healthy controls and patients); **(B)** flow-chart to generate healthy controls’ MNI Atlases (group study); **(C)** assessment method on patients’ cohort. *Only if stated rules apply. **In some cases this operation yielded no results. ^Δ^Four subcortical networks (Anatomical, Picture Naming, Verbal Fluency, Auditory Verb Generation) were used to generate four MNI Atlases; an additional MNI Atlas (“any-task” Atlas) was obtained from the sum of all fMRI-T networks. ^§^Only for AF-SLF and IFOF.

The subject-specific task-specific fMRI-target-ROI obtained from the binary fMRI-ROI was used as a further target-ROI for each Anatomical-T tract to generate task-specific fMRI-T tracts. This latter operation consisted in a “either-end targeting” (a Trackvis built-in function), which allowed to include in the fMRI-T tract only the streamlines whose endpoints are located inside the corresponding fMRI-target-ROI. This pipeline (Figure 1A) produced, for each Anatomical-T tract, as many fMRI-T tracts as the fMRI tasks performed by the subject (Supplementary Figure 2).

### Healthy Controls’ Cohort: Anatomical-T and fMRI-T Atlases (Group Analysis)

For each healthy control, four subcortical networks (Figure 1B) were obtained (Anatomical, PN, VF, AVG) by merging the corresponding tracts (all Anatomical-T, PN fMRI-T, VF fMRI-T, AVG fMRI-T – respectively) using Trackvis built-in “merge” function. Dipy and FSL were then used to generate the four corresponding network-specific density maps and to binarize them with a ≥ 2 threshold (to include only voxels with at least two streamlines). Binary masks were then registered to the MNI space using FSL linear and non-linear registration tools (FLIRT and FNIRT).

Four MNI network-specific Atlases (Anatomical-T, PN fMRI-T, VF fMRI-T, AVG fMRI-T Atlases) were obtained by adding together the binary masks of the corresponding network obtained from all 16 healthy controls, in order to compare the different subcortical networks.

An additional fMRI-T MNI Atlas representing *all* fMRI-T voxels was obtained by adding together the binary masks of all three tasks at the single subject level, and subsequently generating the corresponding MNI Atlas as described for the network-specific Atlases. This additional Atlas is composed by the subject-specific subcortical network underlying all fMRI tasks, and we will refer to it as “any-task” fMRI-T Atlas from now on.

In order to quantify the percentage of each Anatomical-T tract volume included in each fMRI-T tract, a voxel percentage index (VPI) was calculated for each task-specific fMRI-T density map (at the single subject level):

$$\mathrm{VPI}=\frac{\mathrm{number}\,\mathrm{of}\,\mathrm{voxels}\,\mathrm{in}\,\mathrm{fMRI}\,T\,\mathrm{tract}}{\mathrm{number}\,\mathrm{of}\,\mathrm{voxels}\,\mathrm{in}\,\mathrm{corresponding}\,\mathrm{Anatomical}\,T\,\mathrm{Tract}}*100.$$

### Patients’ Cohort: Anatomical-T and fMRI-T Validation Method

For each patient, tumor resection was guided by cortical and subcortical DES evaluating the language function through neuropsychological testing employing naming and counting tasks during awake-surgery. Functional boundaries as intraoperatively identified by DES represented the limit of microsurgical resection. Post-operative 3D-FLAIR was registered to preoperative 3D-FLAIR and subsequently to preoperative B0 using FSL linear registration tool (FLIRT), and the volume of the cavity was segmented using FSL mask tools (cavity-ROI), in order to perform a qualitative analysis and a quantitative analysis (Figure 1C), as follows.

#### Qualitative Analysis

The relationship between the Tractography fascicle models, the limits of resection (corresponding to eloquent structures), and the cavity volume (corresponding to non-eloquent tissue) was evaluated by means of a 3D-rendering of those structures using Trackvis.

#### Quantitative Analysis

For fMRI-T and Anatomical-T AF-SLF and IFOF, a density map of those streamlines was obtained and binarized with a ≥ 2 threshold (to include only voxels with at least two streamlines). These binary masks were then intersected with the cavity-ROI, in order to obtain a ROI representing the voxels belonging to fMRI-T and Anatomical-T AF-SLF and IFOF located inside the surgical cavity. The components of the tracts corresponding to these voxels were considered removed during the procedure, therefore we will refer to them as “removed voxels” for brevity. Such removed voxels were considered as false positive results of Tractography, since they did not correspond to eloquent tissue at DES.

### Patients’ Cohort: Language Function Assessment

Patients’ language function was assessed pre- and post-operatively at several time points using the Milano-Bicocca Battery (Papagno et al., 2012), that included the assessment of semantic and phonemic fluency, speech comprehension, picture naming, and the repetition of words, non-words, and sentences. Median follow-up time was 363 days (range, 6-1158 days), 14 patients out of 16 were evaluated at least for 2 months after surgery, 10 patients out of these 14 were evaluated for more than 12 months after surgery.

### Statistical Analyses

#### Quantitative Analysis on Healthy Controls (VPI Analysis)

Voxel percentage indexes of all the tracts belonging to the same fMRI-T network were compared using Kruskal-Wallis tests and *post hoc* multiple comparisons (Dunn’s tests).

#### Quantitative Analysis on Patients (AF-SLF and IFOF Removed Portions)

“Removed voxels” of AF-SLF and IFOF belonging to different networks (Anatomical-T, PN fMRI-T, VF fMRI-T, AVG fMRI-T) were compared using Kruskal-Wallis tests and Dunn’s non-parametric comparison for *post hoc* testing and Bonferroni correction for multiple comparisons.

#### Clinical Outcome

We retrospectively distinguished patients with long-term (at 12 months) severe deficits and patients that had showed a long-term complete or partial clinical recovery, and compared clinical features of these two groups. Mann-Whitney test was employed to compare continuous numeric variables (age and extent of resection); Fisher’s exact test was employed for categorical variables (sex category, tumor location, preoperative language deficits, and tumor grade – tumor grade was analyzed as a categorical variable, differentiating grade III tumors and tumors of lower grades).

In addition, a linear regression analysis was performed on patients that showed a complete clinical recovery in order to evaluate a correlation between the volume of AF-SLF and IFOF removed portions and the days necessary for the clinical recovery.

## Results

### Healthy Controls’ Tractography Atlases: PN vs VF vs AVG fMRI-T

**FIGURE 2:**
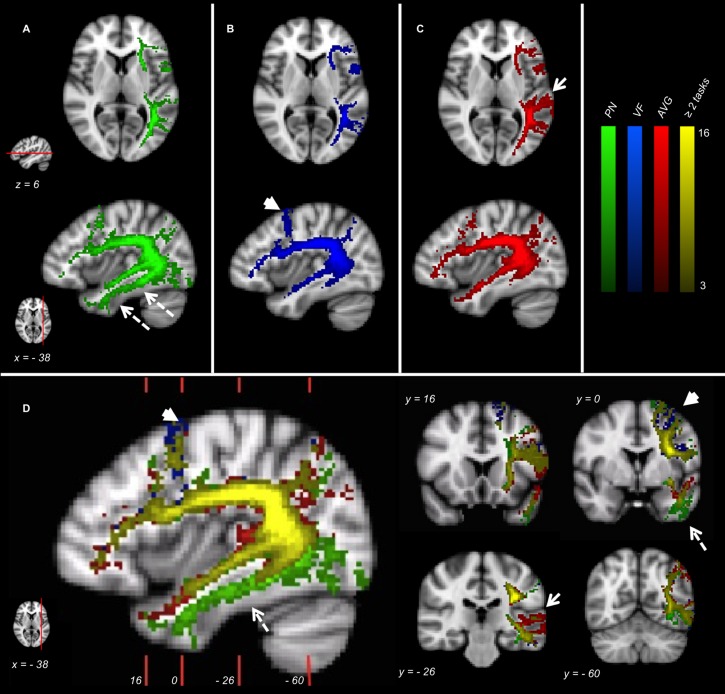
fMRI-targeted Tractography Atlases (group analysis) of 16 healthy controls. **(A)** PN; **(B)** VF; **(C)** AVG; **(D)** Different tasks overlayed. Only voxels represented in at least three subjects are displayed. Color brightness of each voxel is proportional to the number of subjects in whom the voxel was represented. The reference axial and parasagittal MNI slices (MNI *z* = 6, *x* = –38, respectively) are shown on the left side of the figure. The reference coronal MNI slices (MNI *y* = 16, 0, –26, –60) are shown on the parasagittal figure in **(D)**.

The generation of the network-specific fMRI-T Atlases highlighted both task-specific components, and subsets shared by different tasks (Figure 2), as follows.

Picture Naming fMRI-T Atlas involves ventral tracts more than the other tasks. In particular, voxels belonging to ILF are depicted almost exclusively in this Atlas (dotted arrows in Figures 2A,D).

Verbal Fluency fMRI-T Atlas highlights the dorsal stream more than the ventral stream, and some dorsal branches belonging to AF-SLF and FAT are specific to this Atlas (arrowheads in Figures 2B,D).

Auditory Verb Generation fMRI-T Atlas involves temporal subcortical components belonging to AF-SLF more strongly than other tasks (arrows in Figures 2C,D).

A relevant part of the language pathway is shared by different tasks (yellow voxels in Figure 2D), specially the deepest white matter components belonging to AF-SLF and ExC.

### Healthy Controls’ Tractography Atlases: Anatomical-T vs fMRI-T

**FIGURE 3:**
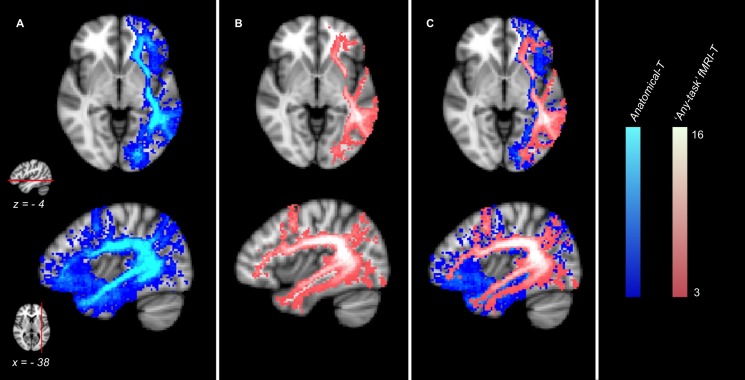
Anatomical and fMRI-targeted Tractography Atlases (group analysis) of 16 healthy controls. **(A)** Anatomical Tractography; **(B)** “any-task” fMRI-targeted Tractography (all tasks included); **(C) (A)** and **(B)** overlayed. Only voxels represented in at least three subjects are displayed. Color brightness of each voxel is proportional to the number of subjects in whom the voxel was represented. The reference axial and parasagittal MNI slices (MNI *z* = –4, *x* = –38, respectively) are shown on the left side of the figure.

The comparison between the Anatomical-T Atlas and the “any-task” fMRI-T Atlas (Figure 3) showed that the fMRI-T network (all tasks included) is a subset of the Anatomical-T network, since several components of Anatomical-T Atlas were not included in the “any-task” fMRI-T one.

### Healthy Controls’ Tractography Quantitative Analysis: Voxel Percentage Index

Voxel Percentage Index analysis revealed that the number of voxels corresponding to each fMRI-T tract trajectory is constantly smaller than the respective Anatomical-T tract, as the mean VPI is always below 60% and, in most cases, is around 40% or even 20%.

**FIGURE 4:**
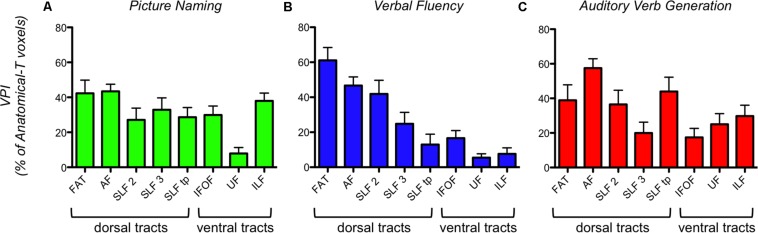
Task-specific VPI (Voxel Percentage Index) of each fMRI-T fiber tract in 16 healthy controls. **(A)** PN; **(B)** VF; **(C)** AVG. The bar graph represents the mean and the standard error.

When comparing VPIs from different tracts within the same task, this analysis showed that different task-specific fMRI-T involve a different percentage of the Anatomical-T corresponding tract (Figure 4).

Verbal Fluency (VF) fMRI-T showed a strong difference between VPIs of dorsal frontal tracts (FAT, AF, SLF-II, SLF-III) and VPIs of ventral (IFOF, UF, ILF) and non-frontal tracts (SLF-tp). This means that a larger percentage of anatomical dorsal frontal tracts reaches VF-related activations, when compared to the percentage of ventral and non-frontal tracts. Kruskal-Wallis test highlighted a statistically significant difference among VPI from all tracts (*P* < 0.0001). *Post hoc* multiple comparisons (Dunn’s tests) revealed that Kruskal-Wallis results are ascribable to a significant VPI difference: between FAT and all ventral and non-frontal tracts; between AF and SLF-tp, AF and UF, AF and ILF; between SLF-II and UF, SLF-II and ILF.

Picture Naming (PN) fMRI-T and Auditory Verb Generation (AVG) fMRI-T did not show a difference between VPIs of dorsal and ventral systems. For PN, Kruskal-Wallis test (*P* = 0.0003) and *post hoc* multiple comparisons (Dunn’s tests) revealed that UF VPIs were significantly lower than VPIs of some other tracts (FAT, AF, ILF). For AVG, Kruskal-Wallis test (*P* = 0.023) and *post hoc* multiple comparisons (Dunn’s tests) showed that AF VPIs were significantly higher than VPIs of some other tracts (SLF-III, IFOF).

### Quantitative Analysis of AF-SLF and IFOF Removed Portions in Brain Tumor Patients

**FIGURE 5:**
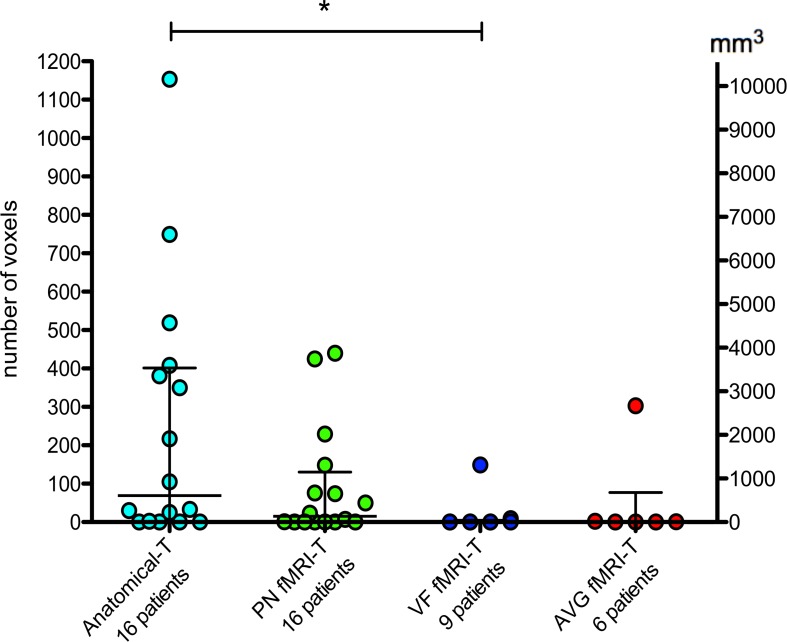
Quantitative analysis of “removed voxels” belonging to AF-SLF and IFOF. The bars represent median and interquartile range. Statistical analysis refers to Table 1. *P < 0.05.

In patients with gliomas, the quantitative comparison between AF-SLF and IFOF removed portions obtained by means of Anatomical-T and task-specific fMRI-T (Figure 5) yielded statistically significant results (Kruskal-Wallis test *P* = 0.035) suggesting that fMRI-T is more specific than Anatomical-T, since fewer non-eloquent voxels (false positive results) belonging to AF-SLF and IFOF were depicted by the fMRI-T approach. *Post hoc* multiple comparisons (Dunn’s non-parametric comparison for *post hoc* testing and Bonferroni correction for multiple comparisons) revealed that Kruskal-Wallis results are ascribable to the significant difference between Anatomical-T and VF fMRI-T (Table 1). Comparing each patient’s removed voxels belonging to Anatomical-T and fMRI-T of AF-SLF and IFOF separately (Supplementary Table 1) reveals several cases for which significant portions of Anatomical-T tracts were resected while their fMRI-T counterparts were completely (or nearly completely) spared. In such cases, a significant number of voxels belonging to the Anatomical-T tract were removed, as opposed to a significantly lower amount of voxels belonging to fMRI-T tracts.

**TABLE 1:** Statistical analysis of “removed voxels” belonging to AF-SLF and IFOF.

					*p-value*^a^		

	Anatomical-T (*n* = 16)	PN fMRI-T (*n* = 16)	VF fMRI-T (*n* = 9)	AVG fMRI-T (*n* = 6)|	Anatomical-T vs PN fMRI-T	Anatomical-T vs VF fMRI-T	Anatomical-T vs AVG fMRI-T
Number of voxels	69	15	0	1	> 0.05	0.033 *	> 0.05
	(1-401)	(0-130)	(0-5)	(0-77)			

Volume of removed voxels (mm^3^)	606	132	0	9			
	(9-3524)	(0-1143)	(0-44)	(0-677)

Note: Data are interpatient median values, with interquartile range in parentheses. ^a^Significant difference between groups. P-values were calculated using Kruskal-Wallis test with Dunn’s non-parametric comparison for post hoc testing and Bonferroni correction for multiple comparisons. *P < 0.05

### Qualitative Evaluation in Brain Tumor Patients: fMRI-T and Anatomical-T vs Limits of Resection

**FIGURE 6:**
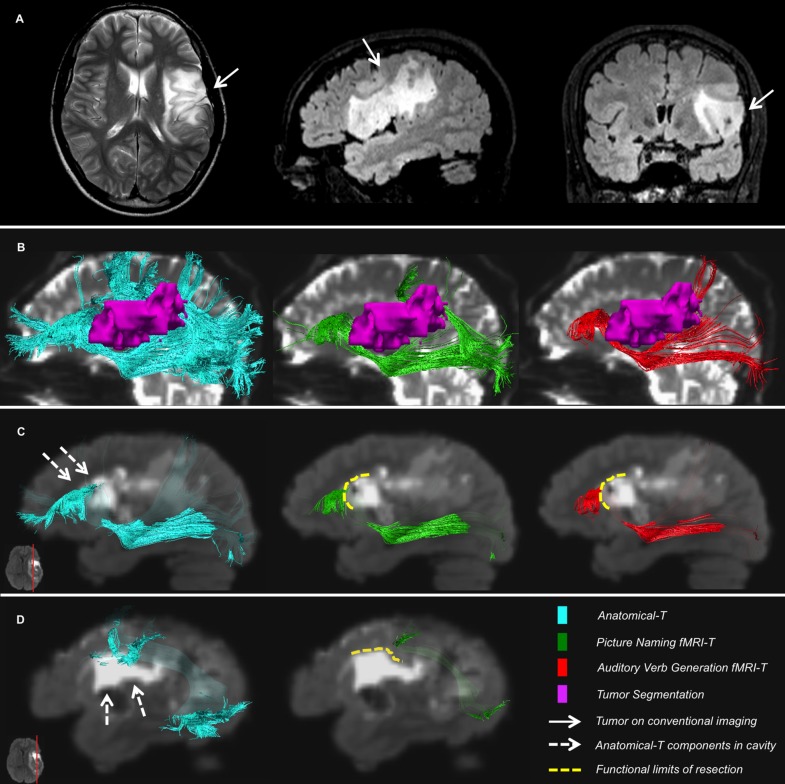
Representative case of a patient. **(A)** T2-weighted and FLAIR images showing the tumor site; **(B)** Relationships between IFOF, AF and the tumor on preoperative B0; **(C,D)** Postoperative FLAIR registered to preoperative diffusion space shows components of Anatomical-T IFOF **(C)** and AF **(D)** located inside the surgical cavity, whereas fMRI-T IFOF **(C)** and AF **(D)** are aligned to the functional limits of resection.

The qualitative evaluation was performed by subdividing the patients’ cohort on the basis of their resection location: patients who underwent a superior frontal resection (eight patients, 50%), patients who underwent a lateral frontal resection (three patients, 18.75%), patients who underwent a temporal pole resection (two patients, 12.5% – in one case the resection also included the insular lobe), patients who underwent a parietal resection (two patients, 12.5% – in one case the resection also included a part of the temporal lobe), one patient who underwent a fronto-parieto-insular resection (one patient, representative case in Figure 6).

#### Superior Frontal Resections

The functional limits of resection corresponded to the trajectory of AF-SLF (to AF in the majority of the cases), and to the trajectory of IFOF [in many cases, more specifically, to the fronto-opercular branch of IFOF – also known as IFOF superficial layer (Caverzasi et al., 2014)]. In a subgroup of these fascicles (four of eight IFOFs, and one out of eight AF-SLFs), VF fMRI-T tracts are aligned to the margins of the cavity, whereas portions belonging to Anatomical-T and PN fMRI-T are located inside the cavity (non-eloquent at DES). Interestingly, as far as IFOF is concerned, in such cases the superior portions of Anatomical-T IFOF and PN fMRI-T IFOF were removed during the procedure, whereas VF fMRI-T IFOF identified exclusively the fronto-opercular portion of IFOF that corresponded to the limits of resection. In all other fascicles but one (three of eight IFOFs, and seven out of eight AF-SLFs) both Anatomical-T and fMRI-T successfully predicted the functional limits of resection. In the remaining case (one IFOF) the procedure removed a portion of IFOF that was common to both Anatomical-T and fMRI-T. As for FAT, different portions of this fascicle were involved by the resection, and there is no clear difference between Anatomical-T and fMRI-T.

#### Lateral Frontal Resections

The functional limits of resection corresponded to the trajectory of AF-SLF (more specifically AF and SLF-III). In one out of three cases, a branch of Anatomical-T SLF-III was involved by the surgery, whereas the trajectory of AVG and PN fMRI-T AF-SLFs were aligned to the margins of the surgical cavity. In two out of three cases, both Anatomical-T and fMRI-T AF-SLFs were able to identify the functional limits of resection.

#### Temporal Pole Resections

The surgical resection was pursued until the deep ExC portion of IFOF (medially) and the temporal components of AF-SLF (more specifically AF and SLF-tp, posteriorly) were encountered. In these two patients, Anatomical-T and fMRI-T equally depicted the eloquent components of these fascicles corresponding to the functional limits of resection. As for UF and ILF, both Anatomical-T and fMRI-T anterior components of these tracts are involved in the surgical resection.

#### Parietal Resections

In these two patients, portions of both Anatomical-T and fMRI-T tracts were removed during the procedure, including AF-SLF and IFOF.

#### Fronto-Parieto-Insular Resection

In this patient, the functional limits of resection are perfectly aligned to AF-SLF and IFOF depicted by PN fMRI-T and AVG fMRI-T, while considerable portions of Anatomical-T tracts are located within the cavity borders (Figure 6).

### Neuropsychological Assessment of Patients’ Language Function

Fourteen patients out of 16 presented with no presurgical language deficit, the remaining two patients presented with a mild deficit (one affecting non-word repetition, the other affecting phonemic fluency and sentence repetition).

Out of 16 patients: four (25%) never experienced post-surgical clinically relevant deficits, five (31.25%) recovered from relevant language deficits within 3 months from surgery day, one (6.25%) recovered within 6 months, one (6.25%) showed a mild language deficit at 12 months (a mild lexicon access and naming impairment), three patients (18.75%) showed severe language deficits at > 12 months (all of the three showed severe lexicon access and comprehension impairment, two of them also showed a naming impairment). For the remaining two patients (12.5%), who showed a language deficit immediately after the procedure, the follow-up only lasted 1 week after surgery, and therefore we were not able to assess their long-term clinical outcome.

The three patients showing severe language deficits at > 12 months were significantly older (Mann-Whitney test *P* = 0.0195) and underwent a less radical surgery in terms of EOR (Mann Whitney test *P* = 0.0195) when compared to the 11 patients showing a complete or partial long-term clinical recovery. A significant statistical association was found between preoperative deficits and long-term severe deficits (Fisher’s exact test *P* = 0.033, positive predictive value 100%, negative predictive value 91.67%). No significant differences were found in tumor grade, tumor location, and sex category between these two groups.

In the 10 patients for whom a complete clinical recovery was documented, the number of days necessary to recover from relevant language deficits after surgery ranged from 0 to 101 days (mean, 42.5; median, 50). A linear regression analysis shows that the number of days necessary to recover from relevant language deficits is directly proportional to the number of AF-SLF and IFOF removed voxels (*P* = 0.043), both when considering Anatomical-T and PN fMRI-T. When considering VF fMRI-T, only two patients out of 10 had VF fMRI-T AF-SLF or IFOF portions removed by the procedure and the clinical recovery time was 78 days for both of them, whereas the mean recovery time for the remaining eight patients without VF fMRI-T AF-SLF or IFOF damage was 33.6 days (all 10 patients performed the VF task). As for AVG fMRI-T, only three patients out of these 10 performed the AVG task; two of them had a mild resection of AVG fMRI-T AF-SLF or IFOF (< 0.02 cc) and experienced no postoperative deficits, the remaining patient had a major resection of AVG fMRI-T AF-SLF or IFOF (∼ 2.67 cc belonging to these tracts) and his clinical recovery time was 78 days.

## Discussion

In this study, we developed a multiparametric pipeline combining Tractography (an anatomy-based technique) with fMRI (a function-based technique) in order to more specifically characterize the classic anatomical Tractography (Anatomical-T). This approach was applied to a healthy controls’ cohort and yielded novel data regarding white matter tracts subserving different language tasks; the subsequent application to a patients’ cohort was useful to highlight those “high-risk” subsets of the anatomical subcortical tracts that are more likely to be eloquent for the language function. This is the first study that systematically combines a HARDI-based Tractography with several language fMRI-tasks for this purpose.

Currently, MR Tractography is the only technique that non-invasively depicts subcortical fiber tracts *in vivo*, which is crucial in the presurgical planning for the resection of brain neoplasms (Bello et al., 2008; Castellano et al., 2012; Essayed et al., 2017). High Angular Resolution Diffusion Imaging (HARDI) acquisitions (Tournier et al., 2004; Tuch, 2004; Hess et al., 2006) have allowed to develop new algorithms that solve complex fiber orientation within the same voxel (Caverzasi et al., 2015), thus depicting subcortical tracts with higher sensitivity (Bucci et al., 2013) and allowing to perform the tracking through tumor-infiltrated areas (Mormina et al., 2016). In particular, the q-ball residual bootstrap probabilistic algorithm (Berman et al., 2008) has shown clinical feasibility, and has recently been employed to fulfill a fiber-tracking protocol that can be routinely applied to depict all major language fascicles in the preoperative setting (Caverzasi et al., 2015).

Nevertheless, intraoperative subcortical direct electric stimulation (DES) remains the gold standard technique to identify fiber tracts trajectory (Berger and Ojemann, 1992; Duffau, 2012; Chang et al., 2015). Although DES and MR Tractography have shown a high degree of concordance (82-97%) (Bello et al., 2008; Leclercq et al., 2010; Castellano et al., 2017), the correspondence is not complete and the main reason is that MR Tractography is not able to provide information about tract functions. In particular, it is believed that some subsets of anatomical language-related fiber tracts may not have an essential function (Mandonnet et al., 2007; Bizzi, 2009). For instance, Bello et al. (2008) clearly reported that selected components of the AF-SLF complex (Superior Longitudinal Fasciculus-Arcuate Fasciculus) do not appear to be eloquent for language tasks when stimulated. This fiber bundle is considered the most relevant language tract; as a consequence, it is reasonable to hypothesize that also other language bundles may include some non-eloquent (or non-essential) subcomponents that can likely be safely removed during the surgical procedure. Providing new tools to preoperatively distinguish the functional core of the language subcortical network from these non-essential subcomponents could be crucial to optimize the presurgical planning of a maximal safe resection of gliomas.

While MR Tractography enables to identify subcortical anatomical structures, data regarding brain cortical structures associated with a specific function can be non-invasively provided by fMRI in the preoperative setting (Talos et al., 2003; Bizzi, 2009; Castellano et al., 2017). In this study, we combine these two techniques in order to achieve a fMRI-targeted Tractography. The possibility of employing Tractography and fMRI in a combined fashion has already been explored by several authors (Conturo et al., 1999; Guye et al., 2003; Dougherty et al., 2005; Smits et al., 2007; Upadhyay et al., 2007; Staempfli et al., 2008; Yang et al., 2009; Kleiser et al., 2010; Saur et al., 2010; Broser et al., 2012; Preti et al., 2012, 2014; Schmitt et al., 2014; Zhu et al., 2014; Figley et al., 2015; Liégeois et al., 2016; Reid et al., 2016). Nevertheless, those studies aimed at using fMRI to quickly and consistently identify the landmarks to be used as a seed for the Tractography algorithm, and the majority of them were performed via DTI-Tractography and exclusively on healthy controls. Conversely, the purpose of the present study is to synergistically combine the two techniques to provide additional information: the depiction of the subsets of the bundles that are connected to language-activated brain cortex. Moreover, this is the first study to develop a systematic pipeline to consistently perform fMRI-based Tractography both on healthy controls and patients with brain tumors, employing an advanced HARDI-based Tractography algorithm (*q*-ball residual bootstrap) and several tasks for language fMRI (picture naming, verbal fluency, auditory verb generation).

We applied this pipeline to a healthy controls’ cohort to provide new data regarding the functional components of the tracts underlying different fMRI tasks.

The comparison among different MNI task-specific fMRI-T Atlases shows both task-specific portions (exclusively included in one Atlas) and common portions (shared by different tasks) of language tracts. Picture Naming fMRI-T Atlas specific voxels are preferentially located within the occipito-temporal ventral stream, and they correspond to ILF trajectory. Although the exact functions of this fascicle are not well defined, its putative role is consistent with this result, since ILF is believed to take part into semantic processing, visual processing, and face recognition (Grill-Spector et al., 2001; Yeatman et al., 2013). Verbal Fluency fMRI-T Atlas specific components belong to AF-SLF and FAT (that constitute the dorsal stream), and voxels belonging to this Atlas are more represented within the dorsal stream rather than the ventral stream. This evidence supports the well defined phonological-articulatory role of the dorsal stream (Rauschecker and Scott, 2009; Friederici, 2012), since Verbal Fluency involves phonological and articulatory capacities more than the other tasks employed, and does not require semantic processing. Auditory Verb Generation fMRI-T Atlas specific portions of the tracts are mainly located within the temporal branch of AF-SLF. As the posterior temporal lobe is crucial for semantic functions (Hickok and Poeppel, 2007), this result is likely due to the strong semantic processing required by Auditory Verb Generation, which is frequently employed to identify temporal areas (i.e., Wernicke area) (Deblaere et al., 2002). A considerable amount of fMRI-T Atlases network is shared by more than one task, and this is compatible with the notion that different tasks partly involve similar language domains: for instance, Picture Naming and Auditory Verb Generation require both semantic and articulatory capacities. More in detail, common voxels shared by several fMRI-T Atlases are preferentially located in the deep white matter, and they correspond to the trajectory of AF-SLF and ExC fascicles. In fact, the trajectories of different language pathway subsets are coherent and located within few voxels at those levels, and this is probably the reason why these deep white matter voxels are shared by two or more task-specific fMRI-T Atlases.

Comparing the Anatomical-T Atlas with the fMRI-T Atlas including all the tasks (“any-task” fMRI-T Atlas) allowed to demonstrate that fiber tracts components whose endpoints are located inside fMRI activations are indeed a subset of the anatomically defined tracts. This means that some other components of language-related tracts have no relationship with fMRI activations derived by any of the tasks, and can therefore be considered as portions of the tract that are not recruited by the language tasks empolyed. This result is consistent with the aforementioned hypothesis [already suggested by other Authors (Bello et al., 2008; Bizzi, 2009)] that some subsets of the language tracts may be redundant, or not strictly related to the language function.

This hypothesis is also supported by the quantitative analysis (VPI analysis) results, since the percentage of the anatomical tracts in relationship with a fMRI activation was always below 60% – and in most cases such percentage was around 40% or even 20%. In addition, VPI analysis clearly demonstrated that Verbal Fluency fMRI-T involved dorsal frontal tracts (FAT, AF, SLF-II, SLF-III) in a significantly higher percentage when compared to the other tracts. This result is consistent with the findings provided by the qualitative MNI fMRI-T Atlas analysis, and supported by the well-defined language dual-stream hypothesis (Friederici, 2012) for the aforementioned reasons. The fact that PN fMRI-T and AVG fMRI-T VPI analysis did not reveal a similar trend is probably due to the fact that these tasks involve several language domains (phonological, articulatory, and semantic at least); whereas Verbal Fluency is almost purely phonological-articulatory.

The same pipeline developed on healthy controls was applied to patients with brain neoplasms, to assess the hypothesis that combining MR Tractography and language fMRI could identify the “high-risk” subsets of language fascicles that are more likely to be eloquent, and should be therefore spared by the surgical resection.

The quantitative comparison between Anatomical-T and task-specific fMRI-T AF-SLF and IFOF reveals that the number of removed voxels (corresponding to non-eloquent points at DES) belonging to Anatomical-T tracts was significantly greater than fMRI-T tracts, and multiple comparison *post hoc* analysis highlights that this difference is due to the difference between Anatomical-T and Verbal Fluency fMRI-T. This result supports our hypothesis that fMRI-T (and specifically VF fMRI-T) is more specific than Anatomical-T when depicting “high-risk” AF-SLF and IFOF components that are eloquent at DES, and therefore should not be removed during the surgical procedure. In fact, fewer non-eloquent voxels belonging to AF-SLF and IFOF were depicted by the VF fMRI-T approach when compared to the Anatomical-T approach, and those non-eloquent voxels are considered false-positive values yielded by the MR Tractography analysis.

The qualitative evaluation of the relationships between language tracts trajectory (Anatomical-T) and DES-defined functional limits of resection revealed a satisfying correspondence between MR Tractography and the limits of resection for AF-SLF and IFOF overall; conversely, some parts of FAT, UF, and ILF trajectories were often located inside the surgical cavity – meaning that those parts did not yield positive responses at DES for the specific intraoperative neuropsychological tests employed. AF-SLF and IFOF are known to be crucial for language function, since they are the fundamental components of the dorsal phonological stream and the ventral semantic stream, respectively (Friederici, 2012). In addition, the preservation of these two fascicles is a crucial objective of brain surgery, as their DES stimulation is known to yield major language disturbances: dysarthria, phonological paraphasias, speech arrest, repetition errors for AF-SLF; semantic paraphasias for IFOF (Bello et al., 2008; Chang et al., 2015). Our analyses indirectly confirm the already well-known match (Bello et al., 2008; Leclercq et al., 2010) between DES and classical Anatomical-T of AF-SLF and IFOF. On the other hand, the importance of preserving FAT, UF, and ILF is not concerted. FAT is a newly defined fascicle (Dick et al., 2014) and recent studies reported that patients experience stuttering (Kemerdere et al., 2016) and speech arrest (Kinoshita et al., 2015) when DES targets this tract. The findings of the present study seem not to confirm such evidences, since in our patients’ cohort the surgical resection including portions of FAT did not lead to long-term language disturbances. UF is considered to participate in the semantic ventral stream (Friederici, 2012), but its role in language function is still not well defined and current literature advocates for no essential language-related functions, except for proper name retrieval (Papagno et al., 2011). As for ILF, recent evidence suggests that this fascicle can be safely removed during surgical procedures, as no language disturbances are evoked by applying DES to its trajectory (Mandonnet et al., 2007). However, some temporal portions of UF and ILF might contribute to some semantic functions (Chang et al., 2015) that were not specifically tested during DES in the present study, even though they were tested before and after surgery.

The qualitative comparison between Anatomical-T and fMRI-T AF-SLF and IFOF, and the evaluation of their relationship with the volume of the surgical cavity, illustrates that in a subset of patients (six out of 16 – 37.5%, in which the resection involved three AF-SLFs and five IFOFs) fMRI-T is able to predict the functional limits of resection *better* than Anatomical-T. In fact, in this subset Anatomical-T tracts are located inside the surgical cavity, whereas fMRI-T ones are perfectly aligned to its margins. In the majority of these cases (four IFOFs), the difference between Anatomical-T and fMRI-T (specifically VF fMRI-T) in predicting the limits of resection is due to the fact that Anatomical-T depicts IFOF superior and anterior frontal branches [also known as IFOF deep layer (Caverzasi et al., 2014)], whereas Verbal Fluency fMRI-T isolates only the component of the fronto-opercular branch (also called IFOF superficial layer) that terminates in a fMRI activated fronto-opercular area. This finding suggests that IFOF superficial layer, that terminates in fronto-opercular language-related cortex, could be the actual language-essential component of this tract. Nevertheless, it should be assessed whether fMRI-T is required to identify the eloquent component, or an anatomy-based target-ROI would be sufficient. In the remaining cases (three AF-SLFs and one IFOF), fMRI-T predicting the functional limits of resection was variously based on all tasks employed, and the surgical procedure involved frontal branches of Anatomical-T AF-SLF and IFOF. However, it should be noted that in a minority of cases some AF-SLF fronto-opercular terminations that were considered non-eloquent for the language function might have a role in praxia and subtle motor skills that were not investigated in the present study. In fact, such functions involve the ventral premotor areas and can be identified by dedicated DES tasks, which were not employed in the present study (Rossi et al., 2018).

Taken together, these analyses demonstrate our hypothesis that fMRI-T may provide additional information about “high-risk” subsets of AF-SLF and IFOF that are more likely to be eloquent, and that fMRI-T based on Verbal Fluency is the most specific for this purpose. Providing fMRI-T high-risk subsets of AF-SLF and IFOF could improve the presurgical planning and, above all, guide intraoperative DES at best, thus further shortening awake-surgery time. In addition, these results provide new data about IFOF, whose superficial layer may be the actual language-related functional core.

Language function assessment confirmed that DES-guided surgical resection succeeded in achieving a safe tumor resection in the majority of patients, who did not suffer from long-term language deficits. In these patients, time to recovery was generally longer when a higher number of voxels representing AF-SLF and IFOF trajectory was involved in the surgical resection (both for Anatomical-T and fMRI-T). This result suggests that DES may be able to predict which fascicle subsets have a role that can be remapped onto other subsets with time, as also previously argued (Duffau, 2012).

As for the subset of patients affected by long-term language deficits, clinical and radiological evidences suggest that several factors may be held responsible of those deficits rather than the surgical resection itself, such as age, preoperative language deficits, disease progression and adjuvant treatment induced toxicity. Other factors possibly impacting the long-term clinical outcome in those three patients were: long-standing previous disease history with multiple surgical interventions (one out of three), preoperative major alterations of language fascicle Tractography (one out of three), postoperative status epilepticus (one out of three), post-operative radiation treatment for residual disease or disease progression (three out of three, one of whom also presented major radiation-induced alterations at MRI scans). Possible factors preventing the complete clinical recovery of the patient showing mild language deficits at 12 months include a long-standing previous disease history with one previous surgical intervention, radiation-toxicity, and a nodule of progressive disease in the territory of the arcuate fasciculus.

This study has some technical and clinical limitations. Firstly, the combination pipeline we developed relies on fMRI-derived binary ROIs. Task-based fMRI sensitivity and specificity strongly depend on the statistical threshold adopted (Roux et al., 2003) and eloquent areas individuation depends on the tasks employed (Black et al., 2017). In this study, we decided to adopt a *P* < 0.001 threshold, instead of a stricter threshold often adopted in the clinical setting (FWE), in order to keep false negative results to a minimum. As for the tasks employed, for the same purpose all but two enrolled subjects were asked to perform at least two different language fMRI tasks. Nevertheless, our battery lacked a syntax-specific task, and therefore this specific domain was not tested. Moreover, awake DES did not employ semantic (Chang et al., 2015), praxis (Rossi et al., 2018) or cognitive (Puglisi et al., 2018) testing. Not testing those specific functions might have contributed to the surgical resection of portions of nervous tissue that had a role in such functions, specifically in the temporal and frontal lobe. Nevertheless, semantic function was extensively tested before and after surgery, and, as already discussed, our results suggest that the surgical resection did not directly result in long-term language disturbances. More in detail, not testing praxis, fine motor skills and other cognitive functions other than language might have caused the surgical resection to include portions of nervous tissue that is non-eloquent for language, but eloquent for other tasks. For instance, praxis involves several cortical areas of the dominant hemisphere, including the oro-facial ventral premotor area (Rossi et al., 2018) that was shown to be adjacent to the language-related fronto-opercular areas (Fornia et al., 2018).

Other limitations are the relatively low number of enrolled patients (specifically, some tumor resection sites have a low number of items), and the fact that data analysis was performed retrospectively. In addition, the evaluation of the relationships between the fascicles and the cavity volume allowed to indirectly infer quantitative data about the specificity of the different Tractography approaches, but did not provide direct measures of their sensitivity – since DES-positive coordinates are not known.

Future studies willing to further assess the advantages provided by fMRI-T may try to overcome the aforementioned limits. Such studies should include both phonological-articulatory and semantic fMRI tasks, as it has also been recently suggested by the American Society of Functional Radiology guidelines (Black et al., 2017). For instance, Verbal Fluency task [which corresponds to Silent Word Generation (Zacà et al., 2013) task in these guidelines], and Verb Generation task [or the Sentence Completion (Zacà et al., 2013) task proposed in these guidelines] could be adopted, respectively. Similarly, a wider intraoperative language assessment, with refined tests for motor cognition and other cognitive functions, could provide a better depiction of functions underlying the tracts herein investigated. Furthermore, in order to obtain robust sensitivity and specificity quantitative data, a greater patients’ cohort should be enrolled, and the exact sites of DES-tested positive and negative voxels should be compared to the trajectory of the fascicles.

Future tractography studies may also employ alternative tracking algorithms that have already been proven feasible on brain tumor patients, such as spherical deconvolution tractography (Mormina et al., 2016; Becker et al., 2019) and multi-fiber tractography (Chen et al., 2015; Gong et al., 2018). These methods, similarly to q-ball tractography, were shown to improve the depiction of complex fiber orientations (Tournier et al., 2007; Malcolm et al., 2010; Fillard et al., 2011; Dell’Acqua and Tournier, 2019). Besides, recent data illustrates how multi-tensor models can increase tracking sensitivity through tissue affected by edema or tumor infiltration by including an additional isotropic tensor reflecting the free water compartment (Gong et al., 2018). Similarly, other multi-compartimental models like NODDI may be employed to improve the tracking of complex fiber configurations (Reddy and Rathi, 2016). Finally, future studies may employ automated or semi-automated approaches for tract selections, such as fiber-clustering, which was shown to consistently group streamlines and assign them to a common cluster, basing on their trajectory similarity (O’Donnell et al., 2017). This approach could overcome the limits of the manual-ROI approach, that can be time-consuming and requires trained operators.

## Conclusion

In this study we develop a systematic pipeline to combine the “classic” Tractography of language fascicles with language fMRI cortical activations in order to achieve a fMRI-targeted Tractography (fMRI-T). Our aim was to provide additional information regarding the functional components of subcortical language fascicles, and to apply this approach both on healthy controls and brain tumor patients.

In healthy controls’ cohort, this approach provided novel insights regarding the subsets of white matter networks related to some fMRI language tasks commonly employed in the clinical setting (Bizzi et al., 2008; Bizzi, 2009). Furthermore, these data are overall consistent with the current theories about language functional neuroanatomy (Hickok and Poeppel, 2007; Rauschecker and Scott, 2009; Friederici, 2012) – e.g., verbal fluency strongly involves dorsal frontal tracts, consistently with the phonogical-articulatory features of this task.

In the patients’ cohort, quantitative and qualitative analyses revealed that this combined method provides additional useful information regarding the “high-risk” subsets of the fascicles that are more likely to be eloquent (specifically AF-SLF and IFOF). Indeed, in some cases fMRI-T predicted the functional limits of DES-guided surgical resection better than Anatomical-T. Employing this novel combined method in addition to the “classic” Anatomical-T could provide additional information to better plan the surgical approach with a refined patient-specific risk-assessment to ultimately guide intraoperative DES and thus resection.

## Data Availability Statement

The datasets for this study are avaliable from the authors upon request.

## Ethics Statement

The studies involving human participants were reviewed and approved by Local ethical committee of Ospedale San Raffaele, Milan, Italy. All subjects provided signed informed consent prior to MR imaging.

## Author Contributions

FS designed the study, acquired, analyzed, and interpreted the imaging data, did the statistical analysis, and drafted the manuscript. EC designed the study, interpreted the diffusion imaging data, and drafted the manuscript. MR and LB acquired the neurostimulation data and drafted the manuscript. KJ designed the study and analyzed the diffusion imaging data. VB and SoC analyzed the fMRI imaging data. PS and AI acquired the imaging data. SaC analyzed the fMRI imaging data and drafted the manuscript. AlC acquired and interpreted the neuropsychological data. AL and GP acquired the data. MG acquired the post-surgical imaging data. MG-T interpreted the imaging data and drafted the manuscript. RH designed the study and interpreted the diffusion imaging data. AF designed the study and interpreted the data. AnC designed the study, acquired and interpreted the imaging data, and drafted the manuscript.

## Conflict of Interest

The authors declare that the research was conducted in the absence of any commercial or financial relationships that could be construed as a potential conflict of interest.

## Footnotes

1 http://www.fil.ion.ucl.ac.uk/spm

2 https://fsl.fmrib.ox.ac.uk/fsl/

3 http://trackvis.org

## Abbreviations

AF: Arcuate Fasciculus
Anatomical-T: Anatomical Tractography
AVG: Auditory Verb Generation
DES: Direct Electrical Stimulation
ExC: External/Extreme Capsule
FAT: Frontal Aslant Tract
fMRI-T: fMRI-targeted Tractography
IFOF: Inferior Fronto-Occipital Fasciculus
ILF: Inferior Longitudinal Fasciculus
MNI: Montreal Neurological Institute
PN: Picture Naming
ROI: Region of Interest
SLF: Superior Longitudinal Fasciculus
SLF-II: II component of SLF
SLF-III: III component of SLF
SLF-tp: temporo-parietal (or vertical) component of SLF
UF: Uncinate Fasciculus
VF: Verbal Fluency.
