# Supplementary material for: fMRI-Targeted High-Angular Resolution Diffusion MR Tractography to Identify Functional Language Tracts in Healthy Controls and Glioma Patients
Source: Front Neurosci. 2020 Mar 31;14:225. doi: 10.3389/fnins.2020.00225 (PMC7136614; doi:10.3389/fnins.2020.00225)
Supplement: Supplementary file 1 [file Data_Sheet_1.pdf]

Supplementary Material

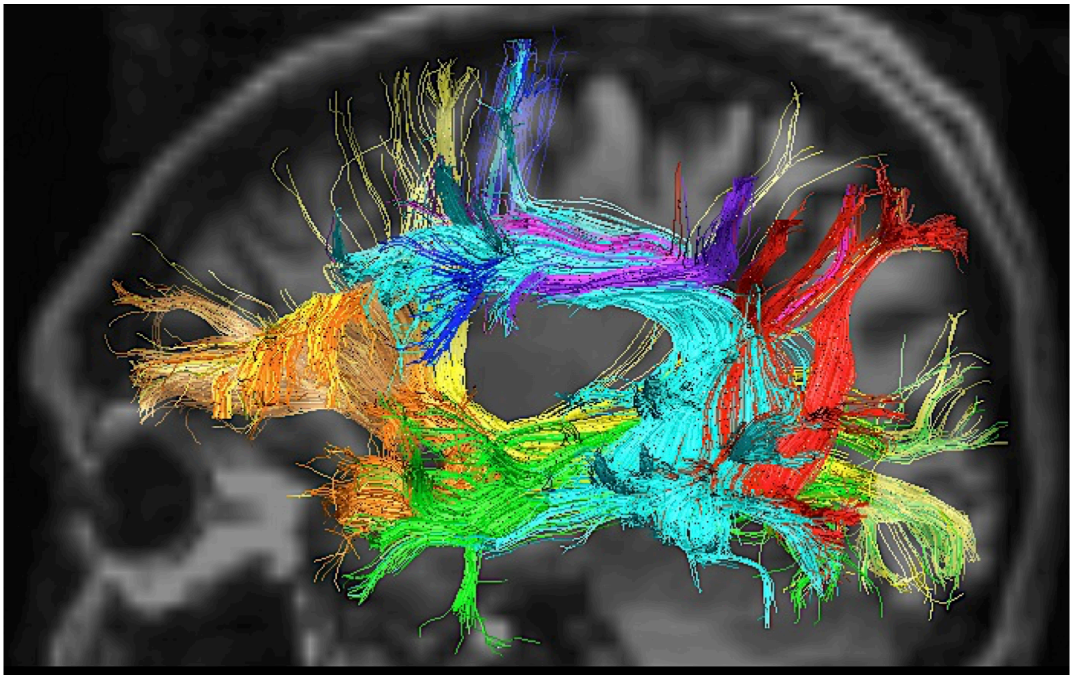

Supplementary Figure 1 – Left-hemisphere language Anatomical-T tracts.
FAT (blue), AF (teal), SLF-II (pink), SLF-III (purple), SLF-tp (red), IFOF (yellow), UF (orange), ILF (green).

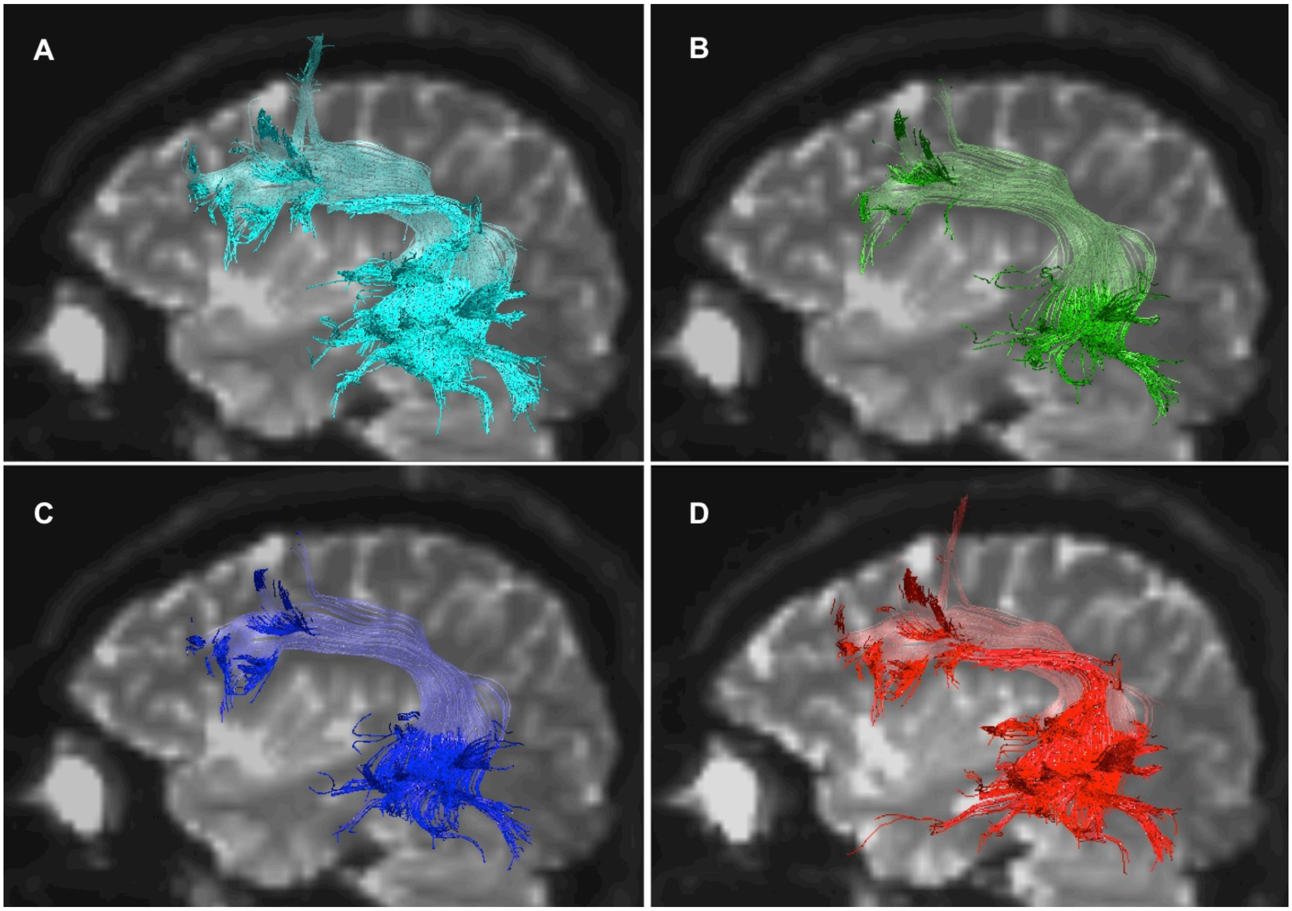

Supplementary Figure 2 – Example of Anatomical-T and fMRI-T of Arcuate Fasciculus (AF) in a healthy control. A) Anatomical-T AF; B) PN fMRI-T AF; C) VF fMRI-T AF; D) AVG fMRI-T AF.

Supplementary Table 1 – Report of 'removed voxels' belonging to AF-SLF and IFOF for Anatomical-T and task specific fMRI-T in each patient.
For each voxel value, the corresponding approximate volume expressed in mm^3 is reported in parentheses. fMRI-T removed voxel values are displayed in bold when remarkably lower than their Anatomical-T counteparts.
PT = Patient number; NP = task was not performed by the patient; NR = the fMRI targeting operation yielded no resuts (in 4 cases out of 5 this was seen when targeting ventral tracts such as IFOF with VF fMRI).

**PT**	**AF-SLF**				**IFOF**			
	Anat-T	PN fMRI-T	VF fMRI-T	AVG fMRI-T	Anat-T	PN fMRI-T	VF fMRI-T	AVG fMRI-T
#01	0 (0)	0 (0)	0 (0)	NP	0 (0)	0 (0)	NR	NP
#02	0 (0)	0 (0)	NP	0 (0)	0 (0)	0 (0)	NP	0 (0)
#03	432 (3797)	74 (650)	NP	NP	96 (844)	**0 (0)**	NP	NP
#04	0 (0)	0 (0)	0 (0)	NP	25 (220)	7 (62)	**0 (0)**	NP
#05	0 (0)	0 (0)	0 (0)	NP	217 (1907)	76 (668)	**0 (0)**	NP
#06	0 (0)	0 (0)	0 (0)	NP	1153(10134)	425 (3735)	**9 (79)**	NP
#07	0 (0)	0 (0)	0 (0)	NP	0 (0)	0 (0)	NR	NP
#08	33 (290)	23 (202)	NP	NP	0 (0)	0 (0)	NP	NP
#09	34 (299)	**0 (0)**	NP	**2 (18)**	71 (624)	50 (439)	NP	**0 (0)**
#10	30 (264)	**0 (0)**	NP	**1 (9)**	0 (0)	0 (0)	NP	0 (0)
#11	0 (0)	0 (0)	0 (0)	NP	749 (6583)	440 (3867)	NR	NP
#12	356 (3129)	201 (1766)	149 (1310)	295 (2593)	168 (1477)	53 (466)	NR	25 (220)
#13	29 (255)	11 (97)	**0 (0)**	NP	352 (3094)	137 (1204)	**0 (0)**	NP
#14	0 (0)	0 (0)	0 (0)	NP	0 (0)	0 (0)	0 (0)	NP
#15	2 (18)	0 (0)	NP	0 (0)	0 (0)	0 (0)	NP	0 (0)
#16	289 (2540)	**0 (0)**	NP	NR	61 (536)	**1 (9)**	NP	**0 (0)**
